# Report of a Case of Gastrostomy

**Published:** 1888-12

**Authors:** Bayard Holmes

**Affiliations:** 125 State Street


### REPORT OF A CASE OF GASTROSTOMY.

BY BAYARD HOLMES, M. D.

Synopsis: Carcinoma (?) of the lower pharynx and upper end of the œsophagus. Advancing stricture of the œsophagus for eight months. Inability to swallow solid food for five months. Dilatation for two months. Inability to swallow liquids for three days. Gastrostomy in two tempos, with an interim of twenty-four hours. No autopsy. Examination of the parts after death showed complete union of the stomach and abdominal wall.

Mr. Reels, 62 years old, a butcher, born in New York, of excellent family history. Father died at 95, mother at 76, years. No consumption in the family. Has two healthy grown-up sons; has always been a well, strong man, and able to carry on his occupation as a butcher without interruption; has had but a single illness. Fifteen years ago, while lifting a barrel of apples into a wagon, he had a copious hæmorrhage from the mouth which greatly weakened him for a few days. Does not know where the blood entered the mouth; had no cough at the time, nor afterward. His usual weight, until six months ago, was between 175 and 200 pounds. Always was a good feeder. Drank only occasionally, and then in small quantities and never to excess.

His present sickness began in December, 1887, with sore throat. This continued uninterruptedly until the last of February, 1888, when it was accompanied by hoarseness, and, later, by aphonia. These symptoms disappeared in two weeks, and an advancing dysphagia took their place. Early in March, he consulted for the first time several physicians, among them, Dr. Mark Lackersteen, who informs me that, after careful examination, he diagnosticated an inflammation of the pharynx, and prescribed some astringent local application. It seems that at that time the neoplasm must have been of very limited extent. By the middle of May, the difficulty in swallowing had so far advanced that that no solid food could be taken, and the patient began to complain of hunger. Rapid loss of strength and weight was noticed. In the early part of July it became necessary to occasionally pass sounds through the stricture, in order that even liquids could be taken. There was never any vomiting or dyspnœa. During sleep there was a little more snoring than usual, but no more than could be referred to loss of strength. The emaciation rapidly advanced during the last two months, until the patient's estimated weight was eighty pounds. The cachexia, though slight, was noticeable. Hunger was always a prominent symptom, and thirst appeared during the last two weeks, as the supply of liquids became limited. Disappointing dreams of feasts aggravated him even in his sleep. At last it became impossible to pass even the smallest sounds. Feeding through a tube was never practiced. There was no very offensive odor from the mouth, nor were there eructations or other disturbances frequently noticed in stricture of the œsophagus.

Dr. Gay Dorn saw the case first on September 13, and I was called in by him on the following day. The patient was extremely emaciated. The skin was dry, and hung on the bones so as to make a remarkable impression on the beholder. It seemed that every trace of adipose tissue was gone, leaving the fossæ of the head exposed. The extremities seemed bloodless, and his pulse was very weak. His voice was not strong, though it would not attract attention from its loss of volume. There was no hoarseness. Weakness was considerable, but not so great as to prevent sitting and walking, as from his bed to a chair. There was a tumor—three inches in diameter—at the beginning of the œsophagus, which seemed attached to the larynx and trachea in front, and moved with movements of these parts. The extreme emaciation favored examination. There were no enlarged lymphatics to be found by palpation. Repeated attempts were made to pass even very small catheters and urethral sounds through the stricture, but in vain. In these attempts the patient was able to offer every assistance, and allowed the guiding finger in the pharynx. This seemed in every way equal to an examination under an anæsthetic, and much safer. The outlet of the pharynx was found closed with a firm ragged mass, which seemed to rise as high as the epiglottis. The slightest touch caused bleeding, which soon stopped. The patient said he was suffering intolerable thirst and great hunger, and asked that a hole be made in his throat so that he could take water. His mouth was dry all the time, and he was troubled occasionally by a muco-purulent discharge which caused coughing, if not removed. Enemas were no longer retained.

Œsophagotomy was not thought advisable, because it is a less tried and more complicate operation, though this was a very favorable case on account of the height of the stricture. Gastrostomy was proposed, and readily accepted by the patient and by the family.

The operation was performed in the patient's residence after considerable attention as to cleanliness. Strict antiseptic precautions were used in the operation. Ether was used as the anæsthetic, though I should prefer chloroform.

Beside Dr. Dorn's, I had the assistance of Drs. Randall, Coe, and Hanna. Beginning directly under the attachment of the cartilage of the eighth with that of the seventh rib, an incision was made upward and inward parallel with the margin of the left costal cartilages and about an inch from them, for an inch and a half. The skin and muscle were divided without discovering any fat. All hæmorrhage was stopped without ligatures. A dense fascia was then carefully divided in the bottom of the wound, and a small opening made in the peritoneum. The abdominal walls were so retracted that a large quantity of air began to rush in. This was first filtered by covering the opening for a moment with a sponge of carbolized gauze. The peritoneal opening was then made continuous with that of the skin, and two fingers introduced to bring up the stomach. I was prepared to find some difficulty in this, but I encountered much more than I had anticipated. The transverse colon lay in the incision. Pushing this down, I felt for the lower border of the liver and the duodenum. These were easily found. Following the gut along toward the stomach, I soon found myself lost on the posterior wall of the abdomen or on the diaphragm. This was repeated several times. At last I enlarged the opening in the same direction toward the right, and succeeded in bringing up what seemed to be the pyloric end of the stomach. The stomach was so much atrophied that it was scarcely four inches long. When, by the coronal artery and the omentum, I had identified the part as the greater curvature and dragged it out toward the left as far as it could be attached to my incision without too much tension, I had an assistant hold up the stomach with a tenaculum forceps and his fingers until I had united again about an inch and a half of the peritoneum and fascia on the left of my incision. Then by means of a silk suture; which did not penetrate the mucous coat of the stomach, I attached to the left angle of the remaining opening a part of the stomach about three-quarters of an inch from the coronal artery and the same distance from the gastro-epiploica. The whole lower edge of the incision was attached to the stomach in the same manner by interrupted sutures. Then, the stomach having been drawn up and out of the incision with a tenaculum forceps and the fingers, the upper edge of the incision was attached to the stomach about half an inch at the farthest from the lower line of sutures. The whole peritoneal edge was then felled down with a fine continuous silk suture. When all was done, an elliptical portion of the anterior wall of the stomach about an inch by half an inch was to be seen, held in place by twenty strong, interrupted sutures. No guides were left for the incision into the stomach. After the muscle and skin at the left angle of the wound were closed over the united peritoneum with a few sutures, the remaining cavity over the stomach was packed with iodoform gauze and dressed.

The patient stood the operation well. It lasted an hour and a quarter. On the following day, with a little anæsthetic, an opening was made into the stomach, the mucous membrane drawn out and stitched to the edge of the wound, and a tube eight millimeters in diameter introduced, through which he was fed. The wound was perfectly agglutinated, and neither suppurated nor caused any pain. Only twice did a fit of coughing cause displacement of the tube and leaking. The capacity of the stomach, which at the beginning was only about four ounces, increased rapidly, until nearly a quart was necessary to satisfy the patient. Such quantities as the capacity of the stomach would admit were given at intervals of two hours, and consisted of milk punches, egg nogg, beef tea, and a liberal quantity of champagne and other stimulants.

In spite of the fact that the patient had great relief and satisfaction from feeding, and called for food and drink as he felt the need of it, experiencing no indigestion, he gradually lost strength, and died at the end of the fifth day. On the fourth day, when the displaced tube was again introduced, the wound was found completely united. After the operation he never complained of any pain or uneasiness that could be referred to peritonitis. The bowels moved regularly after feeding began, except on the last day, when there was diarrhœa. The urine passed voluntarily until the last day, when there was retention, and a catheter was used.

The urine was never examined for albumen, which, I have since learned, appears in starvation. The temperature and pulse record, which was kept by Dr. Dorn, are given :

| DATE. | PULSE. | TEMPERATURE. |
|---|---|---|
| September 14, 3.00 p.m.. | 80 | 98 2-5 Fahr. |
| " 15, 3.30 p.m.. | 75 | 98 2-5 " |
| " 15, 6.30 p.m.. | 108 | 100 2-5 " |
| " 16, 9.00 a.m.. | 100 | 99½ " |
| " 16, p.m....... | 96 | 99 " |
| " 17, a.m....... | 96 | 98 3-5 " |
| " 17, p.m....... | 110 | 99 " |
| " 18, a.m....... | 108 | 98½ " |
| " 18, p.m....... | 120 | 98 3-5 " |
| " 19, a.m....... | 120 | 99 " |

